# Atopic dermatitis and fecundity: a Danish National Birth Cohort study

**DOI:** 10.1093/hropen/hoaf077

**Published:** 2025-12-08

**Authors:** Camilla L Kjersgaard, Anne Gaml-Sørensen, Pernille J Clemmensen, Linn H Arendt, Siri E Håberg, Onyebuchi A Arah, Mette Deleuran, Cecilia H Ramlau-Hansen

**Affiliations:** Department of Public Health, Research Unit for Epidemiology, Aarhus University, Aarhus, Denmark; Department of Public Health, Research Unit for Epidemiology, Aarhus University, Aarhus, Denmark; Department of Public Health, Research Unit for Epidemiology, Aarhus University, Aarhus, Denmark; Department of Public Health, Research Unit for Epidemiology, Aarhus University, Aarhus, Denmark; Department of Obstetrics, Gynecology and Fertility, Regional Hospital Horsens, Horsens, Denmark; Centre for Fertility and Health, Norwegian Institute of Health, Oslo, Norway; Department of Global Public Health and Primary Care, University of Bergen, Bergen, Norway; Department of Public Health, Research Unit for Epidemiology, Aarhus University, Aarhus, Denmark; Department of Epidemiology, Fielding School of Public Health, University of California, Los Angeles (UCLA), Los Angeles, CA, USA; Department of Statistics & Data Science, University of California, Los Angeles (UCLA), Los Angeles, CA, USA; Practical Causal Inference Lab, UCLA, Los Angeles, CA, USA; Department of Dermatology, Aarhus University Hospital, Aarhus, Denmark; Department of Public Health, Research Unit for Epidemiology, Aarhus University, Aarhus, Denmark

**Keywords:** eczema, fertility, time to pregnancy, ART, cohort study

## Abstract

**STUDY QUESTION:**

Do women with atopic dermatitis have higher fecundity than those without?

**SUMMARY ANSWER:**

Pregnant women with a history of atopic dermatitis had a slightly shorter time-to-pregnancy (TTP) and a lower risk of conceiving using infertility treatment than those without.

**WHAT IS KNOWN ALREADY:**

Atopic dermatitis has a characteristic T-helper-2-cell-skewed immune response that mirrors the immune shift in pregnancy, which is necessary for the pregnant woman’s immune response to tolerate the fetus. Therefore, it has been hypothesized that atopic dermatitis may be advantageous for conception and pregnancy maintenance. However, this has not yet been investigated.

**STUDY DESIGN, SIZE, DURATION:**

This cohort study included 88 713 pregnant women from the population-based Danish National Birth Cohort (DNBC), who were enrolled between 1996 and 2002.

**PARTICIPANTS/MATERIALS, SETTING, METHODS:**

The women were defined as having atopic dermatitis if, in a computer-assisted interview conducted around gestational week 17, they reported having ever been diagnosed with atopic dermatitis by a doctor. The women were also asked whether the pregnancy was planned or unplanned, to report their TTP within one of five prespecified categories, and whether they had conceived with the use of infertility treatment. We used multinomial logistic regression models to assess associations between atopic dermatitis and fecundity in seven categories: (TTP <1 month, 1–2 months, 3–5 months, 6–12 months, >12 months, pregnant after infertility treatment or unplanned). In addition, we modeled TTP as dichotomous outcomes using logistic regression: subfecundity (TTP ≥6 months vs <6 months) and infertility (TTP >12 months vs ≤12 months). We also assessed women with an atopic constitution (atopic dermatitis, allergic rhinitis, asthma, and food allergies), as this is associated with more severe atopic dermatitis.

**MAIN RESULTS AND THE ROLE OF CHANCE:**

Overall, 3641 (4.1%) women reported ever having had atopic dermatitis. Compared to women without atopic dermatitis, women with atopic dermatitis had a lower relative risk ratio (RRR) of having a TTP >12 months: 0.83 (95% CI: 0.72; 0.96), and use of infertility treatment, RRR: 0.81 (95% CI: 0.69; 0.94). Moreover, women with atopic dermatitis had a lower odds ratio (OR) of subfecundity: 0.89 (95% CI: 0.82; 0.96) and infertility: 0.85 (95% CI: 0.77; 0.95) than women without in the logistic regression model. Results were robust across several sub-analyses.

**LIMITATIONS, REASONS FOR CAUTION:**

The results can only be generalized to women who eventually conceive, since the DNBC consists only of women who successfully conceived.

**WIDER IMPLICATIONS OF THE FINDINGS:**

The findings support the hypothesis of an immunological benefit in conceiving when having atopic dermatitis. The results are reassuring for women with atopic dermatitis trying to conceive. However, the hypothesis should be further tested in a broader population of women without conditioning on pregnancy.

**STUDY FUNDING/COMPETING INTEREST(S):**

The work was supported by the Danish Council for Independent Research (grant number DFF—1030-00164B), Aarhus University, and Fonden af Fam. Kjærsgaard, Sunds, the Research Council of Norway (project no. 320656), through its Centres of Excellence funding scheme (project no. 262700), and co-funded by the European Union (ERC, BIOSFER, 101071773). However, views and opinions expressed herein are those of the author(s) only and do not necessarily reflect those of the European Union or the European Research Council. Neither the European Union nor the granting authority can be held responsible. This publication is part of the ReproUnion collaborative study, co-financed by the European Union, Intereg V ÖKS (20200407). M.D. has received consulting fees (including participation on advisory boards) from AbbVie, LEO Pharma, Eli Lilly, Incyte, La Roche Posay, NUMAB Therapeutics, Pfizer, Regeneron Pharmaceuticals Inc., Sanofi Genzyme, Almirall, Union Therapeutics, Kymab, and UCB; travel support from LEO Pharma and Sanofi Genzyme; speaker fees from Mustela, Galderma, Leo Pharma, Sanofi Genzyme, and La Roche Posay; Section editor for Journal of the European Academy of Dermatology and Venereology (JEADV). None of these COIs is relevant to the present article. The remaining authors declare that they have no conflicts of interest.

**TRIAL REGISTRATION NUMBER:**

N/A.

WHAT DOES THIS MEAN FOR PATIENTS? Atopic dermatitis is a chronic skin condition that causes dry, itchy, and inflamed skin. Having atopic dermatitis may be beneficial for conceiving, as the immune response associated with atopic dermatitis is similar to the immune response that supports and maintains a normal pregnancy. This study examines whether women with atopic dermatitis become pregnant more easily than women without atopic dermatitis. In a large Danish cohort, women in early-to-mid pregnancy gave information on their history of atopic dermatitis, the time it took for them to get pregnant, and the potential use of fertility treatment. We found that women with a history of atopic dermatitis got pregnant a little faster and needed fertility treatment less often than women without atopic dermatitis. However, the study cannot provide answers about women who never got pregnant since they were not included in the cohort.

## Introduction

Atopic dermatitis is a common chronic inflammatory skin disease that often develops in early childhood, affecting up to 20% of children (Vestergaard *et al.*, 2019). In adults, 2–5% have active disease (Wollenberg *et al.*, 2016), which may significantly reduce the quality of life (Senra and Wollenberg, 2014). The immediate effects of atopic dermatitis, including dry, itchy eczema lesions, a compromised skin barrier function, and sleep disturbances, are well-documented (Silverberg, 2019). However, its long-term implications for reproductive health remain poorly understood.

Although the visible eczema lesions of atopic dermatitis often improve with age, the underlying predisposition may persist, manifesting as dry atopic skin and increased risk of relapses even after long asymptomatic intervals (Weidinger and Novak, 2016; Weidinger *et al.*, 2018; Maintz *et al.*, 2022). Atopic dermatitis is driven by a T-helper (Th) 2-dominant inflammatory response. It is strongly associated with other atopic diseases, such as asthma, allergic rhinitis, and food allergies, partly due to shared genetic factors regulating immune cells and the skin barrier (Ferreira *et al.*, 2017; Silverberg, 2019; Graham and Eigenmann, 2020; Langan *et al.*, 2020; Ravnborg *et al.*, 2021).

In a normal pregnancy, the immune system shifts from a Th1- to a Th2-skewed profile to support implantation and early fetal tolerance (Orefice, 2021). It has been hypothesized that the Th2 dominance seen in individuals with atopic dermatitis may facilitate implantation and early pregnancy establishment, potentially contributing to higher fecundity (the biological capacity to become pregnant; Olsen, 1999), as atopic dermatitis has been associated with reduced risk of spontaneous abortions and increased probability of giving birth at term (Hanzlikova *et al.*, 2009; Isogami *et al.*, 2024). Atopic dermatitis may also positively impact reproductive health through other mechanisms. Up to 50% of individuals with atopic dermatitis have a loss-of-function mutation in the filaggrin gene (*FLG*), a key component of the skin barrier, which is associated with an earlier onset and more severe atopic dermatitis (Bieber *et al.*, 2022). However, FLG loss-of-function has also been associated with enhanced cutaneous absorption of vitamin D (Khatib *et al.*, 2024), which may positively impact reproductive health by improving fecundity (Arab *et al.*, 2019; Jukic *et al.*, 2019). On the other hand, compromised skin barrier and the consequent increased absorption of endocrine-disrupting chemicals and allergens may adversely affect hormonal balance and the reproductive system (Kezic and Nielsen, 2009; Yilmaz *et al.*, 2020; Munera-Campos and Carrascosa, 2024).

Existing research often groups atopic dermatitis with other atopic conditions, such as asthma and allergic rhinitis, into a single exposure category. This approach may obscure condition-specific effects, as different atopic diseases could have distinct influences on fecundity and fertility (Nilsson *et al.*, 1997; Karmaus and Eneli, 2003). However, a Danish study observed that women with allergic rhinitis experienced shorter time-to-pregnancy (TTP) (Westergaard *et al.*, 2003), suggesting higher fecundity. In the present study, we aimed to investigate whether women with atopic dermatitis also exhibit higher fecundity, measured as TTP and less use of infertility treatment, than those without atopic dermatitis.

## Materials and methods

### Ethical approval

The Committee for Health Research Ethics Approval (VEK) in Denmark approved the data collection in the Danish National Birth Cohort (DNBC) (KF 01-471/94), and the Danish Data Protection Agency approved the DNBC (journal number 2012-41-0379). The data handling was approved by Statens Serum Institut (SSI) and is covered by SSI’s general approval (No. 18/04608). The DNBC Steering Committee approved this study (Ref. no. 2020-28), and it is registered by the Danish Data Protection Agency, Aarhus University (2016-051-000001, rec No. 1150). According to Danish legislation, Institutional Review Board approval is not required for register-based research. The participants in this manuscript have given written informed consent to the publication of their case details at enrollment.

### Study population

**Figure 1.**
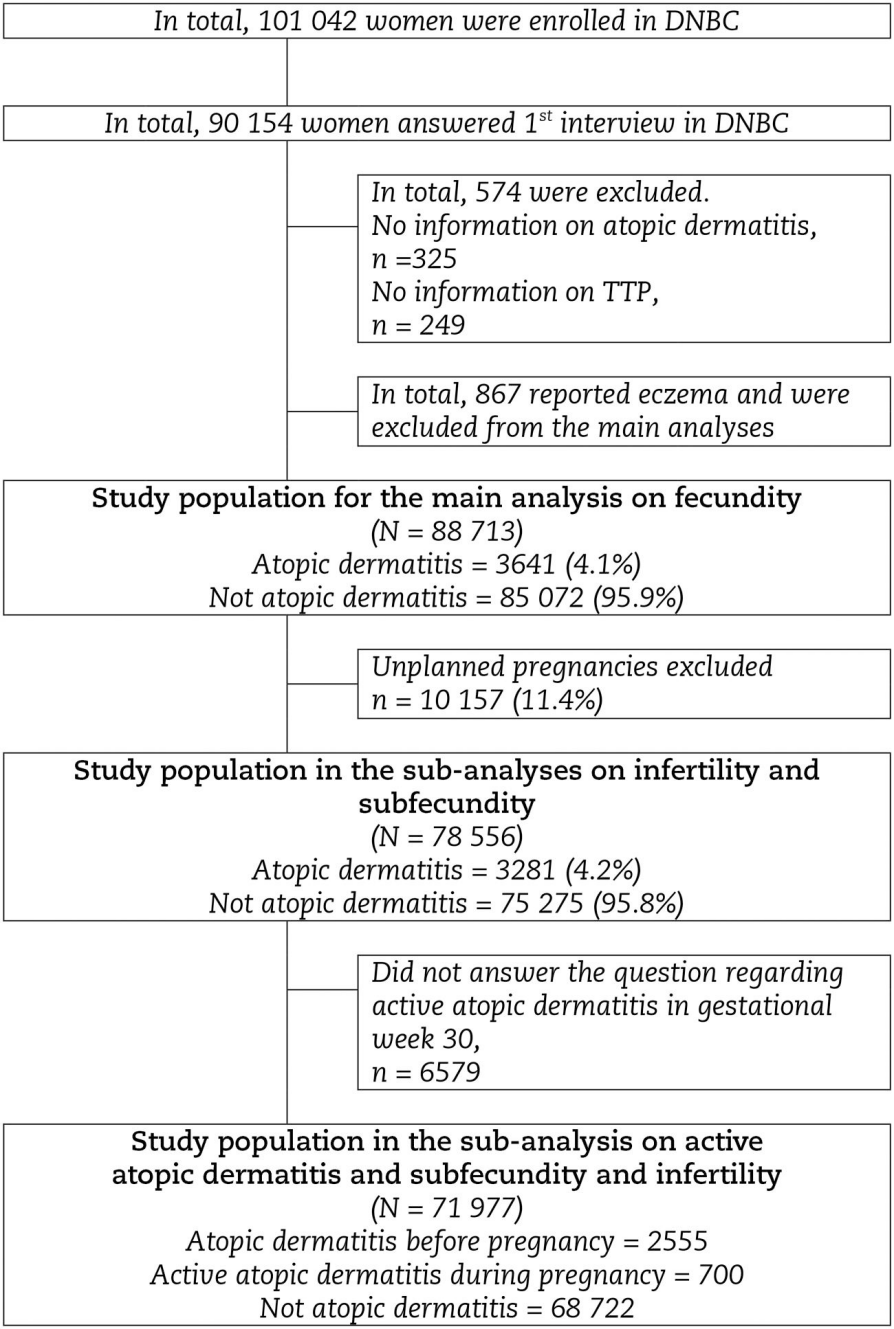
**Flowchart** participants according to atopic dermatitis and fecundity in the Danish National Birth Cohort (DNBC), 1996–2002, Denmark.

This study used data from the DNBC (Olsen *et al.*, 2001; Olsen, 2012), a population-based cohort comprising more than 100 000 pregnancies. Recruitment for the DNBC took place between 1996 and 2002 during the first antenatal visit with the general practitioner (GP). Approximately half of Denmark’s GPs participated in recruiting the women. Around 60% of those invited agreed to participate, and thereby, the DNBC included 30% of all pregnant women in Denmark during the period (Nohr *et al.*, 2006). The women provided information on their lifetime history of atopic dermatitis, lifestyle factors, demographics, health, diseases, TTP, and potential use of infertility treatment twice during pregnancy, at around gestational weeks 17 and 30, using computer-assisted telephone interviews. We excluded 574 women due to missing data on atopic dermatitis and TTP. Women who reported eczema (n = 867) rather than atopic dermatitis were also excluded from the main analysis. Thus, the final study population included 88 713 women (Fig. 1).

### Exposure: atopic dermatitis

The women were defined as having had atopic dermatitis if they, in the first interview in the DNBC, reported ever to have had doctor-diagnosed atopic dermatitis (‘Yes, ever had/have atopic dermatitis’ vs ‘No, never had atopic dermatitis’) (Supplementary File S1). The 867 women reporting eczema, but not doctor-diagnosed atopic dermatitis, were excluded from the main analysis as eczema may or may not reflect atopic dermatitis, introducing diagnostic uncertainty and potential misclassification (Fig. 1). They were included in a sub-analysis.

In a sub-analysis, data from the second interview at gestational week 30 on active atopic dermatitis during pregnancy were used to confirm the exposure and ensure that the women had active atopic dermatitis in adulthood. Moreover, to explore women with an atopic constitution, we utilized information on other atopic conditions: asthma, allergic rhinitis, and food allergies from the first interview during pregnancy, since these conditions share genetic factors with atopic dermatitis and are associated with increased disease severity of atopic dermatitis (Ferreira *et al.*, 2017; Silverberg, 2019). Furthermore, having multiple atopic conditions increases the likelihood that women with atopic dermatitis are classified correctly.

### Outcomes: measures of fecundity

Fecundity was measured as TTP and the use of infertility treatment. From the first DNBC interview, the women were asked whether the pregnancy was planned or unplanned. Women with planned pregnancies were asked to provide their TTP in five prespecified categories. Further, women who answered ‘yes’ to the question: ‘*Did you get any infertility treatment prior to this pregnancy?*’ were placed in the infertility treatment category, regardless of their reported TTP. This study categorized fecundity into seven categories (TTP <1 month, 1–2 months, 3–5 months, 6–12 months, >12 months, pregnant after infertility treatment, and unplanned).

TTP was also analyzed as two dichotomized outcomes, restricted to planned pregnancies, as unplanned pregnancies might represent either the most or the least fecund women (Bonde *et al.*, 2006). Women who conceived following infertility treatment were classified as having a TTP >12 months, in line with Danish regulations requiring at least 12 months of attempted conception before public funding of infertility treatment is granted (Alsbjerg, 2025). We examined two dichotomous outcomes: subfecundity (TTP ≥6 months vs <6 months as the reference) and infertility (TTP >12 months vs ≤12 months as the reference), consistent with previous studies (Guldbrandsen *et al.*, 2014).

### Covariates

We used directed acyclic graphs (Greenland *et al.*, 1999; Pearl, 2010; Cinelli *et al.*, 2024) to identify potential confounding variables (Supplementary Fig. S1). The following variables were included in the statistical model: Socioeconomic status (the highest of the couple), maternal age at birth, pre-pregnancy BMI, lifestyle factors (smoking and alcohol consumption in early pregnancy, for further adjustment for socioeconomic status), and calendar year. Maternal age at birth was obtained from the Danish Medical Birth Registry, while the remaining variables were retrieved from the first interview in the DNBC (Bliddal *et al.*, 2018). See Table 1.

**Table 1.** Baseline characteristics according to atopic dermatitis in 88 713 pregnant women from the Danish National Birth Cohort 1996–2002, Denmark.

	No atopic dermatitis	Atopic dermatitis
n (%)	85 072 (95.9%)	3641 (4.1%)
***Baseline characteristics***		
**Socioeconomic status (highest of the couple), n (%)**		
High-grade professional	20 053 (23.6)	941 (25.8)
Low-grade professional	26 300 (30.9)	1210 (33.2)
Skilled worker	23 863 (28.1)	913 (25.1)
Unskilled worker	12 009 (14.1)	455 (12.5)
Student	1971 (2.3)	100 (2.8)
Economically inactive	876 (1.0)	22 (0.6)
Missing	0	0
**Maternal alcohol in first trimester, n (%)**		
No	47 230 (55.5)	1990 (54.7)
Yes	37 755 (44.4)	1646 (45.2)
missing	87 (0.1)	5 (0.1)
**Maternal smoking in first trimester, n (%)**		
Non-smoker	62 997 (74.1)	2818 (77.4)
0–10 cigarettes/day	18 043 (21.2)	706 (19.4)
>10 cigarettes/day	3822 (4.5)	106 (2.9)
Missing	210 (0.3)	11 (0.3)
**Maternal age at birth (years), mean (SD)**		
Mean age	30.5 (4.3)	30.0 (4.0)
Missing	15	0
**Couple fecundity, TTP, and infertility treatment (months), n (%)**		
<1 month	16 771 (19.7)	767 (21.1)
1–2 months	20 598 (24.2)	958 (26.3)
3–5 months	14 769 (17.4)	658 (18.1)
6–12 months	10 960 (12.9)	461 (12.7)
>12 months	6605 (7.8)	241 (6.6)
Infertility treatment	5572 (6.6)	196 (5.4)
Unplanned pregnancy	9797 (11.5)	360 (9.9)
Missing	0	0
**Food allergy, n (%)**		
Yes	2148 (2.5)	284 (7.8)
No	82 670 (97.2)	3349 (92.0)
Missing	254 (0.3)	8 (0.2)
**Asthma, n (%)**		
Yes	6964 (8.2)	726 (19.9)
No	78 004 (91.7)	2904 (79.8)
Missing	104 (0.1)	11 (0.3)
**Allergic rhinitis, n (%)**		
Yes	12 039 (14.2)	1097 (30.1)
No	72 650 (85.4)	2532 (69.5)
Missing	383 (0.5)	12 (0.3)
**BMI, n (%)**		
Underweight (<18.5)	3780 (4.4)	145 (4.0)
Normal weight (18.5–25)	56 686 (66.6)	2430 (66.7)
Overweight (>25–30)	16 296 (19.2)	687 (18.9)
Obese (>30)	6925 (8.1)	320 (8.8)
Missing	1385 (1.6)	59 (1.6)
**Parity (pregnant with), n (%)**		
First child	39 908 (46.9)	1775 (48.8)
Second child or more	43 802 (51.5)	1816 (49.9)
Missing	1362 (1.6)	50 (1.4)
**Calendar year, n (%)**		
1997–1998	10 912 (96.6)	383 (3.4)
1999	18 265 (96.4)	681 (3.6)
2000	19 167 (96.1)	775 (3.9)
2001	17 513 (95.6)	804 (4.4)
2002	15 814 (95.1)	819 (4.9)
2003	3386 (95.0)	179 (5.0)
Missing	15	0

Percentages may not add up to 100% due to rounding of numbers, due to the General Data Protection Regulation (EUROPEAN-COMMISSION, 2018), GDPR.

TTP, time to pregnancy.

### Statistical analyses

In the main analysis, we applied a multinomial logistic regression model to estimate adjusted relative risk ratios (RRR) with 95% CIs comparing women with atopic dermatitis to women without it, according to fecundity (TTP and infertility treatment), in seven categories. A TTP of 1–2 months was selected as the reference group.

Multivariable logistic regression models were conducted as a sensitivity analysis and for all sub-analyses, with TTP modeled as a binary outcome for both subfecundity and infertility. We estimated odds ratios (ORs) with 95% CIs. An OR <1 indicates a lower likelihood of subfecundity or infertility compared with the reference group of women without atopic dermatitis.

### Sub-analyses

We conducted statistical analyses using STATA 18 (StataCorp LLC, College Station, TX, USA). We conducted three sub-analyses. First, we explored the women reporting active atopic dermatitis in pregnancy and subfecundity and infertility, keeping the group with a history of atopic dermatitis before pregnancy without active symptoms in a separate category. The exposure was categorized as: (i) no atopic dermatitis; (ii) atopic dermatitis before pregnancy; and (iii) active atopic dermatitis during pregnancy. Second, we included the women who reported eczema (n = 867) in the multinomial logistic regression models, as eczema could be atopic dermatitis. The exposure was categorized as: (i) neither atopic dermatitis nor eczema; (ii) atopic dermatitis; (iii) eczema.

Third, we examined women who reported multiple atopic symptoms (e.g. atopic dermatitis, asthma, allergic rhinitis, and food allergies) and infertility (TTP >12 months). The analysis was categorized as: (i) no atopic disease; (ii) only atopic dermatitis; (iii) atopic dermatitis and one other disease; (iv) atopic dermatitis and two or more atopic diseases; and (v) Not atopic dermatitis but other atopic diseases.

Missing data were well below 5% and evenly distributed across exposure categories, suggesting that the risk of bias from complete case analyses is minimal (Lee *et al.*, 2021). All models were fitted using robust variance estimation to account for dependencies between pregnancies by the same individual.

## Results

In total, 88 713 women were included in the study, of which 3641 (4.1%) reported doctor-diagnosed atopic dermatitis (Fig. 1) and 700 had active atopic dermatitis at gestational week 30. Women with atopic dermatitis were, on average, younger, had a higher socioeconomic status, and were more often non-smokers than women without atopic dermatitis. Further, they had a higher prevalence of asthma (20%), allergic rhinitis (30%), and food allergies (8%) than women without atopic dermatitis (Table 1).

**Figure 2.**
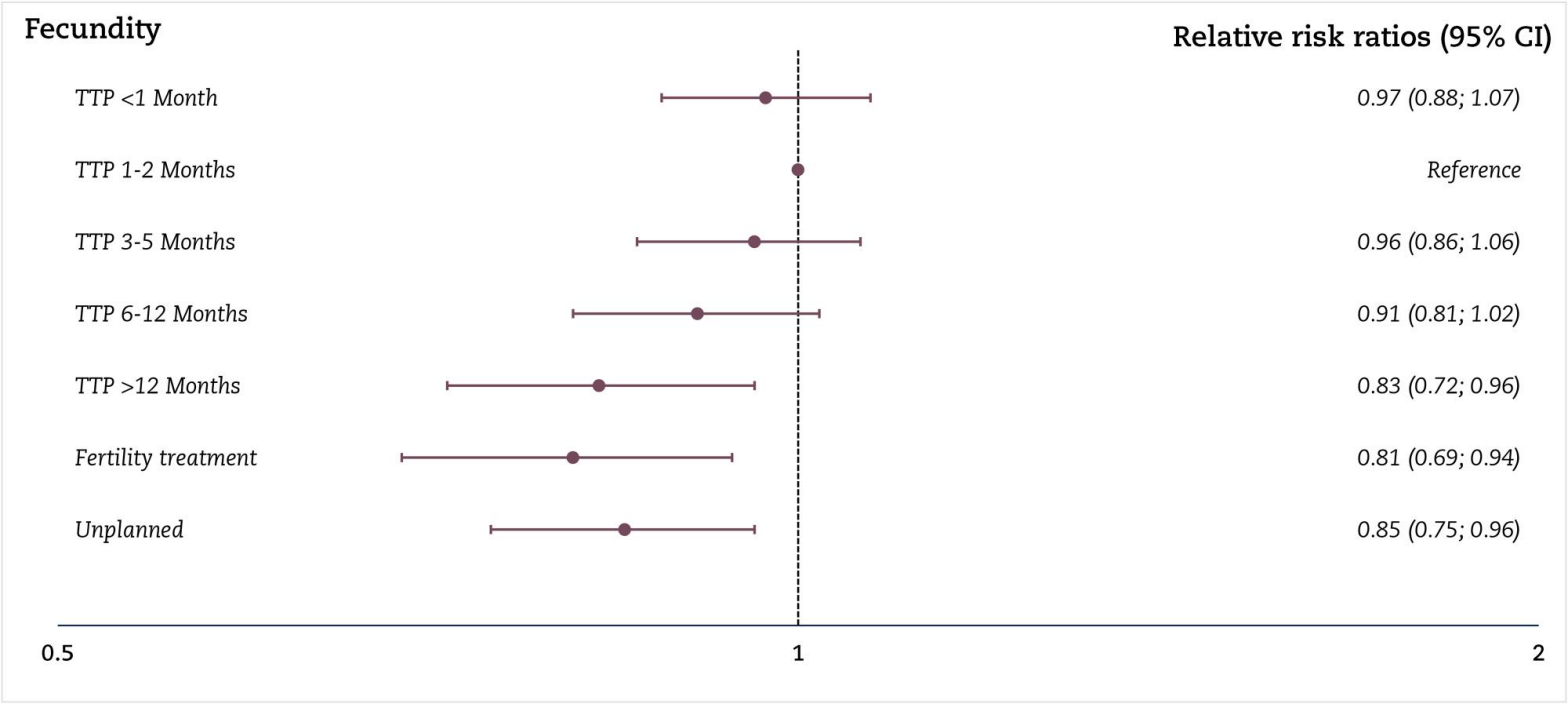
**Main analysis of atopic dermatitis and fecundity**. The multinomial logistic regression according to atopic dermatitis and fecundity in seven categories, with 1–2 months and no atopic dermatitis as the reference, among 88 713 pregnant women, of which 3641 had atopic dermatitis. Results are shown as relative risk ratios with 95% CI. TTP, time to pregnancy. Adjusted for socioeconomic status (highest of the couple), alcohol drinking and smoking in early pregnancy, body mass index, age at birth, and calendar year.

We found that women with atopic dermatitis had a lower risk of the longest TTP categories (6–12 months, RRR: 0.91 (95% CI: 0.81: 1.02) and >12 months, RRR: 0.83 (95% CI: 0.72: 0.96)) and infertility treatment (RRR: 0.81 (95% CI: 0.69; 0.94)) than those without atopic dermatitis. The logistic regression model supported these findings with an OR for infertility of 0.85 (95% CI: 0.77; 0.95) and an OR for subfecundity of 0.89 (95% CI: 0.82; 0.96) in women with atopic dermatitis compared to those without atopic dermatitis (Fig. 2 and Supplementary Tables S1 and S2).

### Sub-analyses

**Figure 3.**
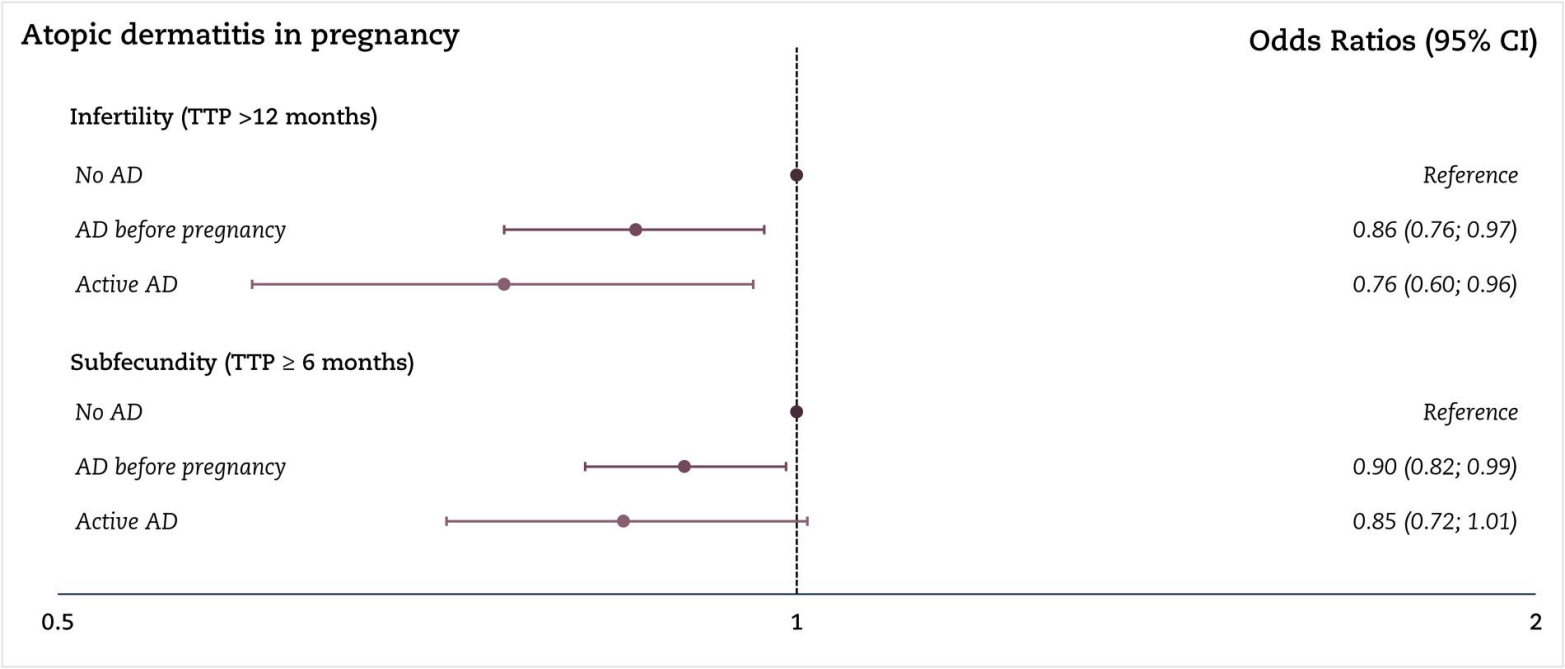
**Sub-analysis of active atopic dermatitis and subfecundity and infertility**. The logistic regression model according to active atopic dermatitis and subfecundity and infertility among 71 977 pregnant women. In total, 2555 women had atopic dermatitis before pregnancy and 700 had active atopic dermatitis during pregnancy. Unplanned pregnancies are excluded, whereas the use of infertility treatment is included in TTP >12 months. In total, 6579 did not answer the question regarding active atopic dermatitis in interview 2 at gestational week 30. Results are shown as odds ratios with 95% CI. AD, atopic dermatitis; TTP, time to pregnancy. Adjusted for socioeconomic status (highest of the couple), alcohol drinking and smoking in early pregnancy, body mass index, age at birth, and calendar year.

**Figure 4.**
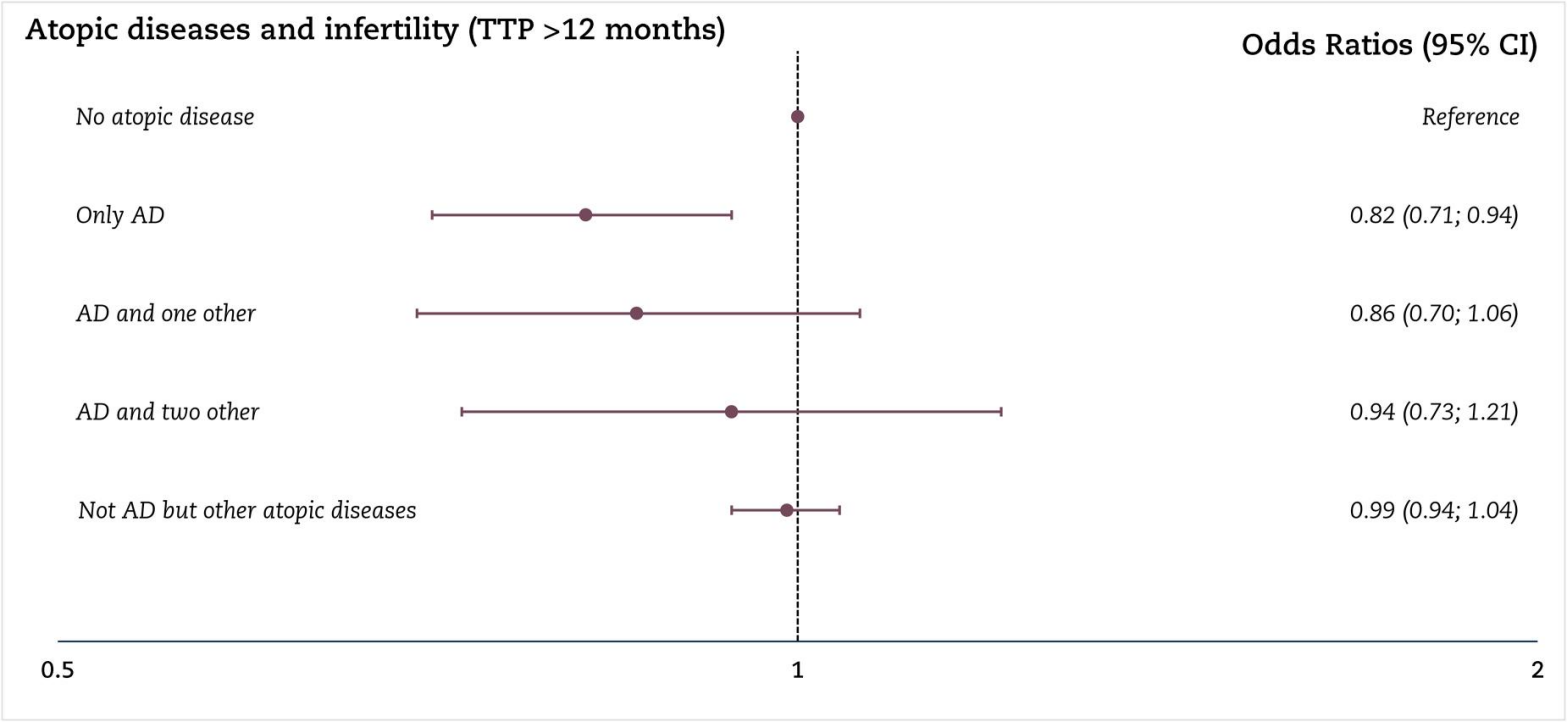
**Sub-analyses of atopic diseases (atopic dermatitis, asthma, allergic rhinitis, and food allergy)**. The logistic regression model according to atopic dermatitis, asthma, allergic rhinitis, and food allergy in a combined variable and infertility among 78 123 pregnant women. Overall, 60 348 (76.8%) had no atopic disease, 1948 (2.5%) had only atopic dermatitis, 815 (1.0%) had atopic dermatitis and one other atopic disease, 496 (0.6%) had atopic dermatitis and two other atopic diseases, and 14 516 (18.5%) did not have atopic dermatitis but had other atopic diseases. Results are shown as odds ratios with 95% CI. AD, atopic dermatitis; TTP, time to pregnancy. Adjusted for socioeconomic status (highest of partners), alcohol drinking and smoking in early pregnancy, body mass index, age at birth, and calendar year. Unplanned pregnancies are excluded from this analysis.

In the first sub-analysis, women who reported active atopic dermatitis during pregnancy had a lower OR of infertility (0.76, 95% CI: 0.60; 0.96) than women without atopic dermatitis; women who had atopic dermatitis but not during pregnancy had an OR of infertility of 0.86 (95% CI: 0.76; 0.97) (Fig. 3). The second sub-analysis, which included women who reported having eczema, showed no association between eczema and fecundity (Supplementary Table S3). In the third sub-analysis of women considered to have an atopic constitution, only women with atopic dermatitis without other atopic conditions had an OR <1 of infertility compared to women without any atopic condition (Fig. 4).

## Discussion

In this large cohort study, we found that women with a history of atopic dermatitis had higher fecundity, including a slightly shorter TTP and a lower likelihood of using infertility treatment. The sub-analyses supported the findings from the primary analysis, and the data analysis on women with active atopic dermatitis during pregnancy showed a stronger association. However, the analysis of atopic constitutions did not show a dose–response relationship with the number of atopic conditions.

We had comprehensive and unique information from 88 713 women in early-to-mid pregnancy about the exposure, outcome, and covariates. The comprehensive self-reported data also allowed us to adjust for multiple important potential confounding variables, thereby reducing the risk of confounding. The self-reported information on doctor-diagnosed atopic dermatitis was a major strength of the study since it allowed us to include women with self-reported atopic dermatitis diagnosed by the GPs or by a private dermatologist. However, with a potential risk of recall bias. The interviews covered a variety of questions on health and lifestyle and did not focus on atopic dermatitis, suggesting that potential misclassification is nondifferential. We excluded women who reported unspecified eczema from the exposure group to avoid misclassification of atopic dermatitis. In a sub-analysis, including the women with eczema, there was no association between eczema and fecundity, likely because the women with eczema encompassed a broad range of skin diseases. We did not include diagnosis codes from the Danish National Patient Register (DNPR), as these are only available for hospital-diagnosed patients in Denmark. Approximately 80% of those suffering from atopic dermatitis experience a mild course of the disease (Ballardini *et al.*, 2013) and will probably never be treated at the hospital and, thereby, never be registered in the DNPR (Lynge *et al.*, 2011). Moreover, we expect that women with a prior diagnosis of atopic dermatitis would report this during the DNBC interview, minimizing the risk of misclassification.

Misclassification of fecundity is a possibility, as TTP reflects couple-level fecundity, not just female fecundity. However, any misclassification is likely non-differential, since it is improbable that the partners of women with atopic dermatitis systematically differ from those of women without atopic dermatitis. There is a potential risk of bias due to recall since participants were asked to remember their TTP in the first trimester. However, Jukic *et al.* (2016) found that women can accurately recall their TTP, though some uncertainty might persist, even when measured shortly after conception. Furthermore, because participants reported TTP categorically, we could not directly calculate fecundability, the probability of conceiving in one menstrual cycle (Eisenberg *et al.*, 2023). Accurate calculations of fecundability require longitudinal TTP data from pregnancy-planner cohorts. In our study, infertility treatment was self-reported, which may have led to some misclassification. A validation study revealed poor agreement between the self-reported infertility treatment and the national IVF register, due to ambiguities in the wording of the DNBC questionnaire. Nevertheless, the self-reported infertility treatment was found to be a valid indicator of sub-fertility (Hvidtjørn *et al.*, 2009), which was the primary focus of the present study. Any misclassification is assumed to be non-differential.

Unplanned pregnancies were included in the main analysis, since it is informative to examine whether women with atopic dermatitis were more or less likely to report unplanned pregnancies. In the present study, 78 556 (89%) of the women reported their pregnancy to be planned, and we observed that women with atopic dermatitis were less likely to have an unplanned pregnancy. Unplanned pregnancies may occur more frequently among both the most and the least fecund women (Bonde *et al.*, 2006). If more highly fecund women without atopic dermatitis are represented in this group, it could explain our results.

The DNBC only includes women who have successfully become pregnant. If atopic dermatitis were to increase sterility or early losses, it would remain undetectable in a study of pregnant women. Consequently, the present study cannot provide insights into women who never conceive. If women with atopic dermatitis experience higher rates of infertility, they may be underrepresented in the study sample, resulting in selection bias. However, exposures that cause sterility will also affect TTP and the use of infertility treatment, and the selection bias will likely only be a theoretical problem rather than a practical one (Olsen, 1999; Weinberg *et al.*, 2021). In our study population, 4% of women had atopic dermatitis, aligning with the prevalence reported by the European Task Force on Atopic Dermatitis/European Academy of Dermatology and Venerology in 2015 for adults (Wollenberg *et al.*, 2016). However, a study on identifying adult atopic dermatitis in questionnaires found that the prevalence varied extensively based on how the question was phrased, and the question, similar to the one asked in DNBC, resulted in the lowest prevalence (Jepsen and Flyvholm, 2007). Since the prevalence found in our cohort matches the estimated prevalence in Europe, we do not expect atopic dermatitis to be strongly associated with participation in our cohort.

The DNBC comprises a higher proportion of women with higher socioeconomic status, which may not directly bias the estimate but could limit its generalizability (Nohr *et al.*, 2006; Jacobsen *et al.*, 2010).

A limitation of the study is the lack of information on the treatment of atopic dermatitis, which would have enabled us to classify the women by disease severity. Consequently, the exposure group probably included mild and severe cases of atopic dermatitis, which could bias our results toward the null. Further, effective treatment of atopic dermatitis could reduce systemic inflammation, thereby minimizing its impact on reproductive parameters (Vakirlis *et al.*, 2024). However, we had data on active atopic dermatitis during pregnancy. We conducted this analysis to explore a higher possibility of increased inflammation before pregnancy, assuming women reporting active dermatitis during pregnancy had more inflammation and a compromised skin barrier function before conceiving, potentially affecting their TTP more substantially than women without atopic dermatitis in adulthood. Some women with atopic dermatitis experience improvement of symptoms during pregnancy, and only 700 of the women in our cohort reported active atopic dermatitis. We observed a slightly stronger association between TTP and women reporting active atopic dermatitis during pregnancy. To investigate the exposure and the hypothesis that Th2 inflammation may confer specific advantages, we conducted sub-analyses including other atopic diseases, as these conditions are associated with severe atopic dermatitis (Silverberg, 2019). The results for atopic dermatitis in combination with asthma, allergic rhinitis, and food allergies showed no association. The group with only atopic dermatitis showed the same results as the main analysis. This may be explained by greater heterogeneity in symptoms and medication use among other atopic diseases, or by the possibility that women with multiple atopic conditions experience more severe disease (Silverberg, 2019; Thyssen *et al.*, 2023), resulting in differences in outcomes between mild and severe atopic dermatitis.

Research on fecundity and fertility in women with atopic dermatitis and other atopic conditions remains limited. In our research, we have previously investigated atopic dermatitis and reproductive outcomes. We found no differences in age in attaining several pubertal milestones among children with atopic dermatitis (Kjersgaard *et al.*, 2025a) and no indication of an adverse effect on reproductive health in young men with atopic dermatitis (Kjersgaard *et al.*, 2025b).

A cohort study by Karmaus and Eneli (2003) examining TTP in women with either atopic dermatitis, allergic rhinitis, or asthma found no significant difference in TTP between women with or without atopic conditions (Karmaus and Eneli, 2003). Three studies have investigated fertility using the total number of children as an outcome; two found slightly higher fertility rates among mothers with atopic dermatitis or other atopic conditions (Nilsson *et al.*, 1997; Karmaus and Eneli, 2003; Tata *et al.*, 2007). In contrast, a more recent cohort study found a strong association between a diagnosis of atopic dermatitis and infertility (Horev *et al.*, 2022). A case-control study by Hanzlikova *et al.* (2009) observed that women with atopic dermatitis were less likely to be in the group experiencing recurrent pregnancy loss, infertility treatment failure, or infertility; although the results were not significant (Hanzlikova *et al.*, 2009). In addition, Karmaus and Eneli (2003) found no difference in rates of spontaneous abortion between women with and without atopic diseases. These discrepancies in the literature may stem from grouping all atopic diseases into a single category, the differences in defining atopic dermatitis, variations in confounder control, and the fact that diagnoses of atopic dermatitis and infertility can be influenced by several external factors, including socioeconomic status and access to healthcare (Andersen *et al.*, 2019; Brautsch *et al.*, 2023; ESHRE, 2023). Together, these limitations highlight the need for further research to better clarify the relationship between specific atopic conditions and reproductive outcomes.

Our findings align with our hypothesis that atopic dermatitis may provide an immunological advantage for conception and enhance our understanding of immune-related factors in fertility and pregnancy. The underlying reasons for these results remain unclear but may involve the Th2-skewed immune system, which could create a unique environment favorable for implanting and supporting early pregnancy. However, this explanation is likely an oversimplification, as a successful pregnancy depends on a complex balance of Th1/Th2 and regulatory T cells/Th17 throughout the pregnancy (Wang *et al.*, 2020). Moreover, the immunopathology of atopic dermatitis is considerably more complex and includes, in the chronic phase, a Th1 activation (Weidinger *et al.*, 2018). It is also plausible that outcomes differ between mild and severe forms of atopic dermatitis. Another potential explanation could be that women with atopic dermatitis had higher levels of Vitamin D. A study found that patients with atopic dermatitis carrying FLG loss-of-function mutations had higher vitamin D levels, and higher Vitamin D levels are associated with higher fecundability, possibly due to uterine receptivity and embryonic implantation (Jukic *et al.*, 2019; Khatib *et al.*, 2024). It is also possible that the observed association is driven by alternative underlying mechanisms or due to bias.

### Conclusion

We found that women with atopic dermatitis had shorter TTP and less use of infertility treatment. These results are reassuring for the large proportion of women with atopic dermatitis. Future studies could further investigate the underlying mechanisms behind these findings. Moreover, the hypothesis should be tested in a population that is not conditioned on pregnancy, ensuring the inclusion of women who never achieve pregnancy.

## Supplementary Material

hoaf077_Supplementary_Data

## Data Availability

The data underlying this article cannot be shared publicly due to national data security legislation on sensitive personal data (General Data Protection Regulation; EUROPEAN-COMMISSION, 2018). However, it could be shared with those who apply at www.dnbc.dk/access-to-dnbc-data.
