# Supplementary material for: Atopic dermatitis and fecundity: a Danish National Birth Cohort study
Source: Hum Reprod Open. 2025 Dec 8;2026(1):hoaf077. doi: 10.1093/hropen/hoaf077 (PMC12802894; doi:10.1093/hropen/hoaf077)
Supplement: hoaf077_Supplementary_Data [file hoaf077_supplementary_data.zip › supplementary_figure_S1_EO.pdf]

Supplementary Figure

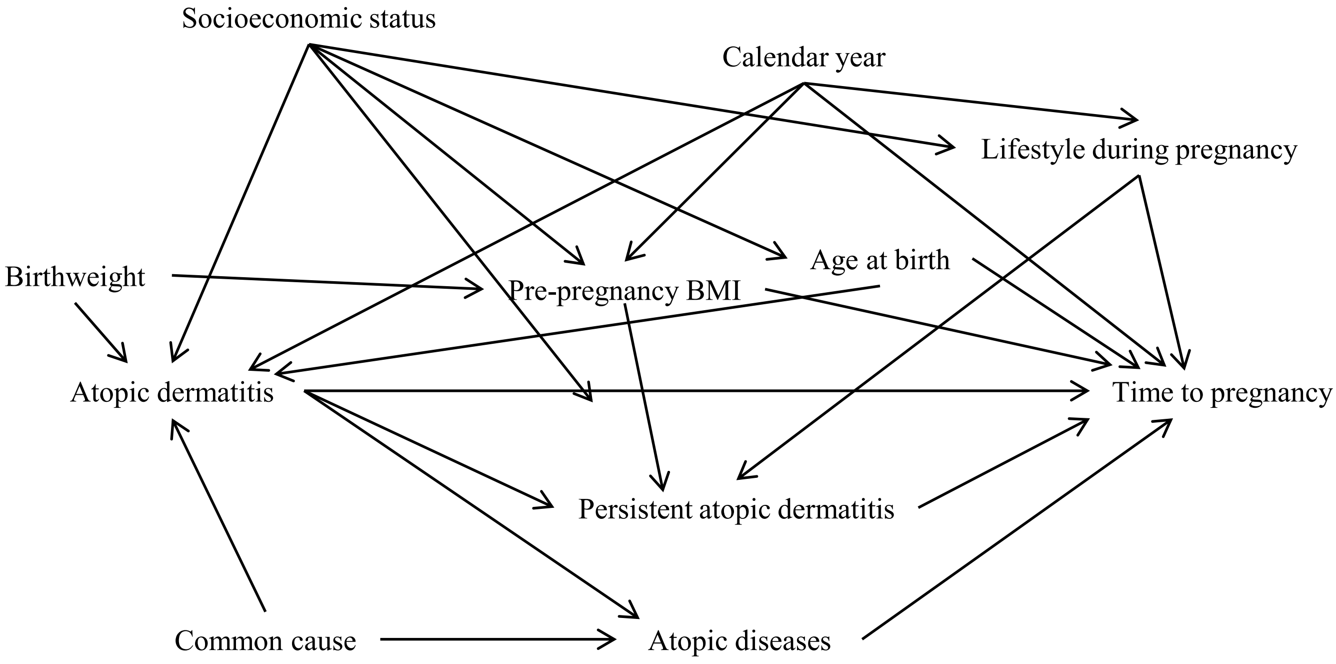

Supplementary Figure S1. Direct acyclic graph (DAG), illustrating the hypothesized causal relationships between atopic dermatitis and fecundity. The DAG shows the causal relationship between atopic dermatitis and fecundity. The following variables were included in all analyses to close any backdoor paths and improve the precision of our estimate: socioeconomic status (highest level of partners), lifestyle during pregnancy (alcohol consumption and smoking in early pregnancy), pre-pregnancy BMI, age at birth, and calendar year.
