# Supplementary material for: Atopic dermatitis and fecundity: a Danish National Birth Cohort study
Source: Hum Reprod Open. 2025 Dec 8;2026(1):hoaf077. doi: 10.1093/hropen/hoaf077 (PMC12802894; doi:10.1093/hropen/hoaf077)
Supplement: hoaf077_Supplementary_Data [file hoaf077_supplementary_data.zip › Supplementary file S1.pdf]

Supplementary file S1: Atopic dermatitis in DNBC:

The exposure variable was made from the following questions: A062: Have you ever had any skin disease? If yes: A063: Was the skin disease diagnosed by a doctor? If yes: A064_6: What kind of skin disease? (answer: Atopic dermatitis).
