# Supplementary material for: Atopic dermatitis and fecundity: a Danish National Birth Cohort study
Source: Hum Reprod Open. 2025 Dec 8;2026(1):hoaf077. doi: 10.1093/hropen/hoaf077 (PMC12802894; doi:10.1093/hropen/hoaf077)
Supplement: hoaf077_Supplementary_Data [file hoaf077_supplementary_data.zip › Supplementary tables.pdf]

Supplementary Tables

Supplementary Table S1. Main analysis of atopic dermatitis and fecundity

	**No** (n = 85 072)	**Atopic dermatitis** (n = 3641)	
		Crude RRR	Adj. RRR (95% CI)
**<1 month**		0.98	0.97 (0.88; 1.07)
**1-2 months**	Reference	1.00	1.00
**3-5 months**		0.96	0.96 (0.86; 1.06)
**6-12 months**		0.90	0.91 (0.81; 1.02)
**>12 months**		0.78	0.83 (0.72; 0.96)
**Infertility treatment**		0.76	0.81 (0.69; 0.94)
**Unplanned**		0.79	0.85 (0.75; 0.96)

The multinomial logistic regression according to atopic dermatitis and fecundity in 7 categories, with 1-2 months and no atopic dermatitis as the reference, among 88 713 pregnant women. Results in relative risk ratios (RRR). Adjusted for Socioeconomic status (highest of the couple), alcohol drinking and smoking in early pregnancy, BMI, age at birth and calendar year.

Abbreviations: Adj: Adjusted; RRR: Relative risk ratio.

Supplementary Table S2. Sensitivity analysis of subfecundity and infertility

	No	Atopic dermatitis	
		Crude OR	Adj. OR (95% CI)
**Infertility**			
TTP ≤12 months	Reference	1.00	1.00
TTP >12 months		0.80	0.85 (0.77; 0.95)
**Subfecundity**			
TTP <6 months	Reference	1.00	1.00
TTP ≥6 months		0.85	0.89 (0.82; 0.96)

The logistic regression model according to atopic dermatitis and subfecundity and infertility, among 78 556 pregnant women (unplanned pregnancies are excluded, whereas infertility treatment is included in TTP >12 months). Adjusted for Socioeconomic status (highest of couple), alcohol drinking and smoking in early pregnancy, BMI, age at birth and calendar year.
Abbreviations: Adj: Adjusted; OR: Odds ratios.

Supplementary Table S3. Sub-analysis of atopic dermatitis, eczema, and fecundity

	No	Atopic dermatitis		Eczema	
		Crude RRR	Adj. RRR (95% CI)	Crude RRR	Adj. RRR (95% CI)
**<1 month**		0.98	0.97 (0.88; 1.07)	1.18	1.17 (0.96; 1.44)
**1-2 months**	Reference	1.00	1.00	1.00	1.00
**3-5 months**		0.96	0.96 (0.86; 1.06)	1.14	1.15 (0.93; 1.42)
**6-12 months**		0.90	0.91 (0.81; 1.02)	1.10	1.03 (0.82; 1.31)
**>12 months**		0.78	0.83 (0.72; 0.96)	1.07	1.08 (0.81; 1.43)
**Infertility treatment**		0.76	0.81 (0.69; 0.94)	0.95	0.96 (0.70; 1.32)
**Unplanned**		0.79	0.85 (0.75; 0.96)	1.01	1.00 (0.77; 1.29)

The multinomial logistic regression according to atopic dermatitis and eczema in two separate groups and fecundity in 7 categories, with 1-2 months and no atopic dermatitis as the reference, among 89 580 pregnant women. Results in relative risk ratios (RRR). Adjusted for Socioeconomic status (highest of couple), alcohol drinking and smoking in early pregnancy, BMI, age at birth and calendar year.

Abbreviations: Adj: Adjusted; RRR: Relative risk ratios.
